# Using Vaccine Safety Data to Demonstrate the Potential of Pooled Data Analysis

**DOI:** 10.3390/vaccines12091052

**Published:** 2024-09-14

**Authors:** Steven Hawken, Lindsay A. Wilson, Kumanan Wilson

**Affiliations:** 1 Clinical Epidemiology Program, Ottawa Hospital Research Institute, 1053 Carling Avenue, Ottawa, ON K1Y 4E9, Canada; shawken@ohri.ca; 2 School of Epidemiology and Public Health, University of Ottawa, 600 Peter Morand Cres., Ottawa, ON K1G 5Z3, Canada; 3 School of Population and Public Health, University of British Columbia, Vancouver, BC V6T 1Z3, Canada; 4 Bruyere Research Institute, Ottawa, ON K1R 6M1, Canada; 5 Department of Medicine, University of Ottawa, Ottawa, ON K1H 8M5, Canada; 6 CANImmunize Inc., Ottawa, ON K2A 2E5, Canada

**Keywords:** adverse events following immunization, COVID-19, pooled data, self-controlled case series, vaccine safety

## Abstract

In Canada, vaccine safety studies are often conducted at the provincial/territorial level where the primary data on vaccination reside. Combining health services data from multiple jurisdictions using a pooled data analytic approach would reduce the amount of time needed to detect vaccine safety signals. To determine the difference in the time it would take to identify safety signals using different proportions of the Canadian population, we conducted power and sample size calculations for a hypothetical self-controlled case series-based surveillance analysis. We used scenarios modeled after the real-world examples of myocarditis and vaccine-induced immune thrombotic thrombocytopenia (VITT) following COVID-19 vaccination as our base cases. Our calculations demonstrated that in the case of a myocarditis-type event, a pooled analysis would reduce the time needed to detect a safety signal by over 60% compared to using Ontario data alone. In the case of a VITT-type event, a pooled analysis could detect a safety signal 49 days sooner than using Ontario data alone, potentially averting as many as 30 events. Our analysis demonstrates that there is substantial value in using pan-Canadian health services data to evaluate the safety of vaccines. Efforts should be made to develop a pan-Canadian vaccine data source to allow for an earlier evaluation of suspected adverse events following immunization.

## 1. Introduction

The rollout of COVID-19 vaccines was among the largest public health interventions in Canadian history. Although the rollout was extremely successful and resulted in the vast majority of the population receiving at least one vaccine dose, the pandemic also highlighted the need for better health data and information systems that can enable real-time assessments of vaccine safety, efficacy, and coverage. These metrics are critical for post-market surveillance and evaluations of the effectiveness of immunization programs [1].

The pandemic highlighted the value of having large sample sizes and real-time data to conduct rapid evaluations of vaccine safety. This was particularly relevant given the emergence of unexpected adverse events following immunization (AEFIs), including vaccine-induced immune thrombotic thrombocytopenia (VITT) and myocarditis. While pooling health services data across jurisdictions can provide the larger sample sizes needed to detect these rare adverse events quickly, there are political and data challenges to achieving this objective.

### Our Approach

In order to demonstrate the potential value of adopting pooled health services data for vaccine safety surveillance in Canada, we considered two representative simulated scenarios informed by evidence-based clinical data and population demographics. The goal of this study was to determine how much more quickly a pooled data analytic approach could identify vaccine safety signals for each scenario compared to provincial and territorial data alone, allowing earlier mitigation strategies to be applied and potentially avoiding significant harm.

## 2. Materials and Methods

### 2.1. Scenario Assumptions

Tables illustrating our underlying assumptions regarding the Canadian population by province and age group are presented in the Appendix. These tables illustrate the number of individuals in the total Canadian population, the pediatric population, and the population of adults over the age of 65, as well as the number of expected adverse events that would occur in each of these populations across a range of vaccine acceptance rates (30%, 50%, and 90%) and adverse event rates (1/100, 1/1000, and 1/10,000). These three population groups allow us to consider how many individuals may be eligible for vaccination and how many people may therefore be at risk of an AEFI. The number of individuals in each of these groups is drawn from the 2021 Canadian census [2]. The sample vaccination rates were chosen to reflect scenarios with very high vaccine acceptance (e.g., childhood polio vaccines; >90% uptake) [3], moderate vaccine acceptance (e.g., pneumococcal vaccine among older adults; ~55% uptake) [4], and low vaccine acceptance (e.g., seasonal influenza vaccine; 30–40% uptake) [5]. Finally, the adverse event rates selected allow us to simulate the number of adverse events that would occur in each of these populations and at each of these hypothetical vaccination rates for events that are common or events that are rare. All of these factors impact the number of vaccine doses and the amount of time that would be needed to detect a safety signal.

To illustrate these assumptions in a simulated real-world scenario, we conducted two base-case analyses, myocarditis following vaccination with mRNA vaccines and VITT following receipt of the ChAdOx1 nCoV-19 vaccine. We chose these scenarios because in the case of myocarditis, the uncertainty about the existence and magnitude of the risk impacted the public perception of the vaccine, and in the case of VITT, the risk impacted the decision on who would receive the vaccine.

For the first analysis, we chose a scenario representing myocarditis following COVID-19 vaccination. Myocarditis has a population incidence of approximately 1 in 100,000, and surveillance studies and randomized controlled trials (RCTs) have suggested that there is a three-fold increase in the risk of myocarditis following COVID-19 vaccination (relative incidence = 3.0) [6]. Our self-controlled case series (SCCS) design specified an observation window of 180 days proximal to vaccination and a risk period including the 28 days immediately following the receipt of a COVID-19 vaccine dose [7]. Power and sample size requirements for SCCS studies are driven by total events rather than total subjects, so the total sample size required is then determined based on the expected event rates for AEFIs of interest [8,9] (given the population incidence of myocarditis, the expected event rate for this analysis was set at 1 in 100,000). We set the desired statistical power at 90% (the probability of correctly rejecting the null hypothesis in favor of an effect being present), and the two-sided alpha at 5% (the desired significance level of statistical tests and calculation of 95% confidence intervals) [8,9].

For the second analysis, we presented a scenario based on VITT following vaccination with a ChAdOx1 nCoV-19 vaccine. VITT was not observed during clinical trials of this vaccine and remained very rare, but published epidemiological studies reported that the incidence of VITT after vaccination with ChAdOx1 nCoV-19 ranged between 1 case per 26,000 vaccinations and 1 case per 127,000 vaccinations, and was much rarer in other vaccine types (from 1 in 500,000 to 1 in 1,000,000) [10]. The Medicines and Healthcare products Regulatory Agency (MHRA) in the UK reported a VITT rate of 15.1 per 1000,000 first doses of ChAdOx1 nCoV-19 based on the administration of 41 million first doses of the vaccine [11]. The vast majority of VITT cases occurred following the first dose, and 80% of these events occurred among women between the ages of 20 and 55 [11]. Hence, our base-case analysis assumed an incidence rate of 15 VITT events per 1 million vaccine doses administered (1.5/100,000) and an effect size of relative incidence (RI) = 5.0, as reported in Higgins et al. [11]. Higgins et al. [11] used an observation window of 90 days with a 24-day risk interval following vaccination exposure in their VITT analysis. However, for clarity and consistency with our myocarditis base case, we used the same risk interval of 28 days and an observation window of 180 days with 90% power and two-sided alpha = 5%, which led to only minor differences in the results. VITT results are presented both overall and for males and females aged 18–39. We were unable to report results specifically in females aged 20–55 years to reflect the MHRA findings, as we were limited by the age- and sex-stratified population estimates available from Statistics Canada.

All power and sample size estimates used in our reported findings were calculated using the SCCS package in R [8,12], which provides the number of observed events required based on formulas published by Farrington and colleagues based on the specified relative incidence, observation windows, and alpha (0.05) and power (90%) [8].

### 2.2. Analyses for Other Specific Scenarios

We have provided calculations from two focused examples describing the potential benefits of conducting pooled analyses for vaccine safety surveillance. However, given the diversity of assumptions underlying different surveillance scenarios, we have also developed a web application based on our underlying algorithms that can be used to calculate results for a range of assumptions, including effect size (relative incidence), adverse event rates, vaccine program throughput, and different combinations of provincial and territorial population sizes [13]. This will enable the reader to calculate results for any desired set of assumptions not specifically reported in this manuscript.

## 3. Results

### 3.1. Scenario 1: Myocarditis

Based on our scenario assumptions for the base case of myocarditis, 47 events would need to be observed overall in the study window to detect a safety signal. If we assume that a mass vaccination campaign in Ontario could achieve a throughput of 50,000 vaccine doses administered per day [14], we would expect to observe 47 myocarditis events in Ontario after 94 days (47/(50,000 * 0.00001)). However, if health services data from multiple jurisdictions could be combined, assuming a similar vaccination throughput, the amount of time needed to detect this event would decrease substantially (Table 1).

### 3.2. Scenario 2: Vaccine-Induced Immune Thrombotic Thrombocytopenia (VITT)

To detect an effect based on our model assumptions for VITT, 20 VITT events would need to be accrued. Taking the example of Ontario, if a total throughput of 50,000 vaccinations per day was achieved [14], a total of 27 days would be required to accrue sufficient events. However, as the risk was particularly high among individuals between the ages of 18 and 39 (roughly 30% of the eligible Ontario population), only 15,811 vaccines would be administered per day among individuals in this age range, requiring 85 days of accrual to obtain the necessary number of events. If we further restrict our analyses to women between the ages of 18 and 39 (i.e., the group at highest risk of VITT following vaccination), this would correspond to about 7500 vaccines administered per day. To detect a safety signal in this case, 178 days of accrual would be required.

However, if a pooled analysis was conducted that included males and females between the ages of 18 and 39, the number of accrual days needed would be substantially reduced (Table 2). Compared to the 85 days required to accrue the necessary number of events using Ontario data alone, combining health services data from patients in this age group from Ontario and Quebec would require only 56 days. Combining health services data from British Columbia (BC), Alberta, Saskatchewan, Manitoba, Ontario, and Quebec would enable the identification of a safety signal 49 days sooner than using Ontario data alone, possibly avoiding as many as 28 events if intervention occurred following the timelier pooled analysis.

**Figure 1:**
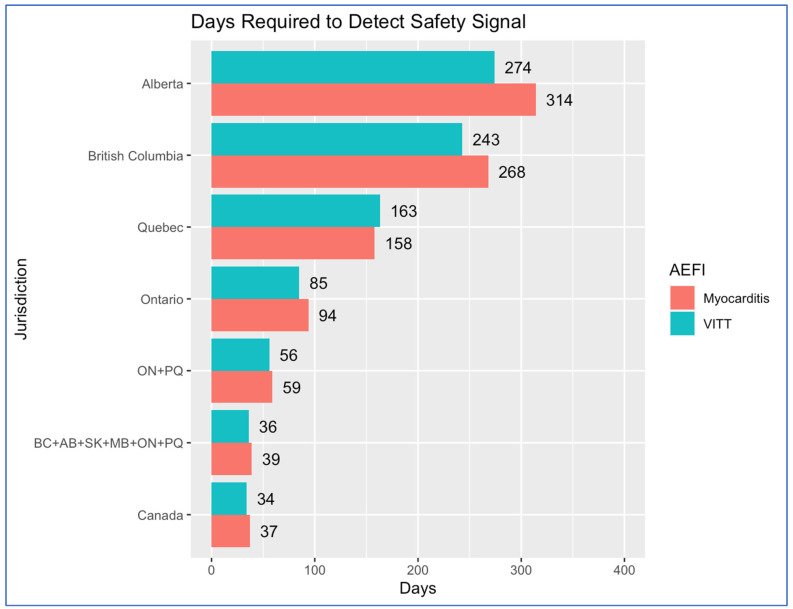
Days of mass vaccination throughput required to accrue sufficient events to detect: (1) myocarditis assuming relative incidence (RI) = 3.0, incidence = 1/100,000 in overall population, and (2) vaccine-induced immune thrombocytopenia and thrombosis assuming RI = 5.0, incidence = 1.5/100,000 in M/F 18–39 yrs. Assumptions: Power: 90%; two-sided alpha: 0.05; risk period: 28 days; observation window: 180 days. The same relative throughput achieved in Ontario has been assumed to be possible in other jurisdictions (~0.35% of the eligible population/day). Abbreviations: AB, Alberta; BC, British Columbia; MB, Manitoba; ON, Ontario; PQ, Province of Quebec; SK, Saskatchewan.

The overall trends identified in Table 1 and Table 2 are summarized in Figure 1. However, myocarditis and VITT are just two examples of the adverse events that could be observed following vaccination. In Table 3, we present the number of days that would be required to accrue the necessary number of events for a more common adverse event (event rate of 1/10,000) and an extremely rare event (event rate of 1/1,000,000), given the same SCCS design and assumptions. In all cases, adverse events could be detected much faster if health services data could be drawn from across the country.

## 4. Discussion

The scenarios modeled in our analysis suggest that the use of pooled data analytics would enable more rapid identification of vaccine safety signals than individual provincial or territorial data alone. This, in and of itself, is not surprising. However, our analyses help to quantify this impact and its potential relevance for public health.

In the case of a post-mRNA vaccine myocarditis-type scenario, compared to using Ontario data alone, a pooled analysis would reduce the amount of time needed to detect an event by over 60% (94 days for Ontario alone vs. 37 days for a Canada-wide analysis). The same message holds for rarer or more common adverse events, with rates of 1/1,000,000 and 1/10,000. Especially for very rare adverse events, an important safety signal can be detected substantially faster when surveillance draws on a larger proportion of the total population. This is especially important when it comes to very severe adverse events, where reducing the amount of time required to detect the safety signal could have life-saving implications.

In our analysis of a VITT-like scenario, our findings suggested that a safety signal could be detected in just over one month using a pooled approach, compared with nearly three months using Ontario sdata alone. This is a particularly striking example of how combining health services data can help to identify rare events that disproportionately impact certain populations. In the real world, younger women were much more likely to develop this rare but serious adverse event. Given that younger women comprise a relatively small proportion of the population, in the absence of combined data, this signal could not be identified until a very large number of doses had been administered. Had the safety signal been detected earlier, as many as 28 cases of VITT could have been averted, corresponding to as many as 6 deaths, given the estimated fatality rate of VITT (~23%) [15].

Of note, this analysis was intended to determine the value of a pooled health services data system for a future event similar to myocarditis and VITT following vaccination against COVID-19. During the actual COVID-19 vaccine rollout, some data regarding VITT risk after vaccination with the Astra-Zeneca vaccine were already available in other jurisdictions. These data did ultimately influence the decision on who would receive the vaccine in Canada. Our analysis was developed in anticipation of a future scenario where Canada receives novel vaccines around the same time as other jurisdictions. In a real-time and real-world scenario, vulnerable subgroups of vaccinated individuals (e.g., younger females), exact risk windows, effect sizes, and other assumptions would not be known in advance, hence the need to consider a variety of plausible scenarios when planning for future surveillance.

As more vaccines are approved for use in humans, strengthened monitoring of adverse events and maintaining robust safety data will be essential for protecting public health and public trust. Traditional Phase 3 trials are often inadequately powered to detect rare adverse events, which can lead to serious consequences for patients, undermining public trust and fueling waves of disinformation [16]. During the roll-out of mRNA vaccines for COVID-19, post-market surveillance revealed that the incidence of myocarditis was higher than expected, particularly among young people. In British Columbia, the rate of myocarditis within 7 days of vaccine administration was 0.97/100,000 vaccine doses, compared to an expected rate of 0.13/100,000 [17]. Subgroup analyses revealed that rates were even higher among young males, leading to changes in the recommended vaccine dosage for this population [18].

Similar delays in the detection of adverse events occurred during the roll-out of the H1N1 influenza vaccine in 2009–2010. After a rapid vaccine rollout in response to the 2009 influenza pandemic, clinicians in Finland and Sweden detected an increase in the incidence of narcolepsy among children and adolescents who received the pandemic vaccine, an association that was later detected in numerous other European countries [19]. Evidence from Sweden indicated that the incidence of narcolepsy among children and adolescents aged 4–19 years was 4.2/100,000 among vaccinated patients compared to 0.64/100,000 in the unvaccinated cohort. This risk was not identified during the clinical trial phase and was not detected until over 30 million vaccine doses had been administered [19].

Drawing on lessons learned from previous pandemics, active surveillance was also adopted to monitor cases of Guillain–Barré Syndrome (GBS) during the 2009–2010 swine flu pandemic. GBS, an acute inflammatory demyelinating polyneuropathy, was identified as a rare but serious adverse event following national immunization efforts in the United States during the 1976 influenza pandemic. Concerns over the increased incidence of GBS ultimately contributed to the cessation of influenza immunization campaigns in the United States at that time [20]. Studies during the 2009–2010 influenza season found a much lower attributable risk of GBS from vaccination than during the 1976 pandemic, and suggested that the risk of GBS from vaccination was lower than the risk of GBS from influenza illness [21]. Nonetheless, this active surveillance was essential for ensuring that safety signals could be detected as early as possible and measures could be taken to mitigate the risk of adverse events.

These real-world examples offer significant insights into the importance of robust, high-quality adverse event surveillance initiatives. In all of these cases, meaningful increases in the incidence of severe adverse events could not be detected until hundreds of thousands (and even millions) of vaccine doses had been administered, a number that could never be attained in a clinical trial setting. Thus, ensuring that large amounts of post-market surveillance data can be brought together is essential for the timely detection and response to safety signals that emerge.

The improved detection of adverse events across demographic subgroups also has important implications for health equity. Many adverse events are not evenly distributed across the population and may have more severe impacts on vulnerable populations. Drawing on data from only one province or territory increases the risk of missing safety signals among vulnerable or priority populations due to a lack of statistical power. Similarly, as demographics vary by region, so too do vaccine lot distributions. Analyzing data from only one jurisdiction risks overlooking lot-specific adverse events. By combining health services data from across the country, the capacity to detect these signals more quickly, even for rare events, is increased. These sizable reductions in the amount of time needed to detect effects would enable the more rapid deployment of mitigation strategies and help to prevent ongoing morbidity and mortality by alerting clinicians to potential risks. Importantly, it would also allow public health officials to more effectively communicate and characterize risks to vaccine recipients. In the absence of data, anti-vaccine communities have the opportunity to distort and magnify perceived risk through the spread of misinformation.

### 4.1. Limitations

Although all measured and unmeasured baseline (fixed) confounders are implicitly controlled with the SCCS design, this design does not control for time-varying confounders (e.g., transient exposures that vary within individuals during the observation period) [8]. While this is an important limitation for the SCCS design, this limitation is true of other study designs as well (e.g., cohort and case–control study designs). However, the most common time-varying exposure, age, can be accounted for in an SCCS analysis. Age stratification can be incorporated into a standard SCCS analysis, or a non-parametric implementation of the SCCS can be applied to account for age-related changes in baseline risk across the observation period. Age is especially important to consider when the observation period is very long and the risk for the event of interest changes according to age. The scenarios we have described in this report are unlikely to be susceptible to age effects due to the relatively short observation periods, but this may be an important consideration for future studies.

Our study is also based on the assumption that a vaccination rollout would be consistent across regions and time. In reality, vaccination rates are unlikely to be consistent over time and across subgroups, and are likely to drop as population interest declines. We also assumed similar incidence rates across provinces, which may not be the case. These limitations in our analysis further speak to the value of having pan-Canadian health services data where more accurate and granular data on these variables would be available.

An additional limitation that may arise is the risk of bias due to the fact that people who receive vaccines are generally healthy, which may conceal the presence of effects within the days immediately following vaccination. Again, this also presents a risk of bias in other study designs, which would result in a differential bias between vaccinated and unvaccinated individuals where unexposed (unvaccinated) individuals were included.

Finally, despite the potential value of a pooled data system, there are challenges associated with any analytic approach that relies on combining data from multiple jurisdictions. One such obstacle is the need for standardized immunization data across Canada. Data quality at the provincial/territorial level is also a limitation, as vaccine registries may not have real-time data and may therefore need to rely on hybrid data sources (administrative data plus registry data).

### 4.2. Implications

Our analyses demonstrate the value of leveraging health services data from populations in multiple jurisdictions across a variety of safety scenarios. While our analysis pertained to safety, similar inferences could be drawn for effectiveness, particularly given the capacity to conduct test-negative designs using health administrative data.

The importance of robust post-market surveillance is heightened by the advent of a multitude of novel vaccines for a variety of demographic groups. Furthermore, the mRNA platform creates new challenges by allowing for a rapid iteration of vaccines to address antigenic drift. As occurred during the COVID-19 pandemic, this can result in the release of updated vaccines without Phase 3 trial data, relying on post-market surveillance to verify safety and effectiveness. Strengthened post-market surveillance will be critical for adapting to these new approaches to vaccine development.

However, challengeston this approach includd the willingness of jurisdictions to share data, the lack of data in jurisdictions, and the standardization of data. To address concerns about data ownership, federated approaches to analyses are increasingly being considered and employed. These have been used in Canada for evaluations of drug safety and for international vaccine safety surveillance [22,23]. A federated approach could have substantial value in the Canadian setting and pave the way to more rapid and robust vaccine safety analyses in the future.

## 5. Conclusions

Our calculations demonstrate that in the case of a myocarditis-type event, a pooled analysis would reduce the time needed to detect a safety signal by over 60% compared to using Ontario data alone. In the case of a VITT-type event, a pooled analysis could detect a safety signal 49 days sooner than using Ontario data alone, potentially averting as many as 30 events.

Given the potential offered by pooled data analysis for Canada and the importance of post-market surveillance efforts, there is substantial value in the development of a pooled/pan-Canadian approach. As our analysis has demonstrated, this approach, by leveraging a larger sample size, can enable more rapid assessments of vaccine safety. Furthermore, by combining health services datasets from across multiple jurisdictions without the need for datasets to be removed from their secure environments, these analyses can be conducted while still maintaining data security and patient privacy. This approach would allow policymakers to develop robust, evidence-based policies that may prevent substantial morbidity and mortality while maintaining confidence in vaccines.

## Figures and Tables

**Table 1:** Days of mass vaccination throughput required to accrue sufficient events to detect a relative incidence of 3.0 for myocarditis.

Province/Territory	2021 Population	Throughput (Vaccinations/Day)	Events/Day 1/100,000 Risk	Days to Accrue Necessary Events
Ontario	14,223,942	50,000	0.50	94
Quebec	8,501,833	29,885	0.30	158
British Columbia	5,000,879	17,579	0.18	268
Alberta	4,262,635	14,984	0.15	314
Manitoba	1,342,153	4717	0.05	997
Saskatchewan	1,132,505	3980	0.04	1181
Nova Scotia	969,383	3407	0.03	1380
New Brunswick	775,610	2726	0.03	1725
Newfoundland and Labrador	510,550	1794	0.02	2620
Prince Edward Island	154,331	542	0.01	8672
ON + PQ	22,725,775	79,885	0.80	59
BC + AB + SK + MB + ON + PQ	34,463,947	121,145	1.21	39
Canada	36,991,981	130,028	1.30	37

Assumptions: Population risk for myocarditis: 1/100,000; power: 90%; two-sided alpha: 0.05; risk period: 28 days; observation window: 180 days. The same relative throughput achieved in Ontario has been assumed to be possible in other jurisdictions (~0.35% of the eligible population/day). Abbreviations: AB, Alberta; BC, British Columbia; MB, Manitoba; ON, Ontario; PQ, Province of Quebec; SK, Saskatchewan.

**Table 2:** Days of mass vaccination throughput required to accrue sufficient events to detect a relative incidence of 5.0 for vaccine-induced thrombotic thrombocytopenia in males and females aged 18–39 years.

Province/Territory	2021 Population (Aged 18–39)	Throughput (Vaccinations/Day)	Events/Day 1.5/100,000 Risk	Days to Accrue Necessary Events
Ontario	4,517,570	15,811	0.24	85
Quebec	2,337,880	8182	0.12	163
British Columbia	1,568,044	5488	0.08	243
Alberta	1,395,268	4883	0.07	274
Manitoba	429,186	1502	0.02	888
Saskatchewan	351,071	1228	0.02	1086
Nova Scotia	273,295	956	0.01	1395
New Brunswick	199,309	697	0.01	1913
Newfoundland and Labrador	126,163	441	<0.01	3024
Prince Edward Island	47,631	166	<0.01	8033
ON + PQ	6,855,450	23,993	0.36	56
BC + AB + SK + MB + ON + PQ	10,599,019	37,094	0.56	36
Canada	11,287,640	39,506	0.59	34

Assumptions: Population risk for vaccine-induced thrombotic thrombocytopenia: 1.5/100,000; power: 90%; two-sided alpha: 0.05; risk period: 28 days; observation window: 180 days. The same relative throughput achieved in Ontario has been assumed to be possible in other jurisdictions (~0.35% of the eligible population/day). Abbreviations: AB, Alberta; BC, British Columbia; MB, Manitoba; ON, Ontario; PQ, Province of Quebec; SK, Saskatchewan.

**Table 3:** Days of mass vaccination throughput required to accrue sufficient events to detect a relative incidence of 3.0 for hypothetical adverse events.

Province/Territory	Days to Accrue Necessary Events	
	1/1,000,000 Event Rate	1/10,000 Event Rate
Ontario	940	10
Quebec	1573	16
British Columbia	2674	27
Alberta	3137	32
Manitoba	9964	100
Saskatchewan	11,810	119
Nova Scotia	13,796	138
New Brunswick	17,242	173
Newfoundland and Labrador	26,199	262
Prince Edward Island	86,716	868
ON/PQ	589	6
BC/AB/SK/MB/ON	516	6
Canada	362	4

Assumptions: Power: 90%; two-sided alpha: 0.05; risk period: 28 days; observation window: 180 days. The same relative throughput achieved in Ontario has been assumed to be possible in other jurisdictions (~0.35% of the eligible population/day). Abbreviations: AB, Alberta; BC, British Columbia; MB, Manitoba; ON, Ontario; PQ, Province of Quebec; SK, Saskatchewan.

## Data Availability

All analyses in this manuscript are based on publicly available data from the 2021 Canadian census.

## References

[1] Government of Canada (2023). Canadian Immunization Registry Functional Standards 2020 to 2024: Recommendations from the Canadian Immunization Registry and Coverage Network. https://www.canada.ca/en/public-health/services/publications/vaccines-immunization/canadian-immunization-registry-functional-standards-2020-2024.html.

[2] Government of Canada SC (2022). Profile Table, Census Profile, 2021 Census of Population—Canada. https://www12.statcan.gc.ca/census-recensement/2021/dp-pd/prof/index.cfm?Lang=E.

[3] Government of Canada (2023). Highlights from the 2021 Childhood National Immunization Coverage Survey (cNICS). https://www.canada.ca/en/public-health/services/immunization-vaccines/vaccination-coverage/2021-highlights-childhood-national-immunization-coverage-survey.html.

[4] Government of Canada (2022). Vaccine Uptake in Canadian Adults 2021. https://www.canada.ca/en/public-health/services/immunization-vaccines/vaccination-coverage/highlights-2020-2021-seasonal-influenza-survey/full-report.html.

[5] Government of Canada (2022). Seasonal Influenza Vaccination Coverage in Canada, 2021–2022: Full Report. https://www.canada.ca/en/public-health/services/immunization-vaccines/vaccination-coverage/seasonal-influenza-survey-results-2021-2022/full-report.html.

[6] Ontario Agency for Health Protection and Promotion (Public Health Ontario) (2022). Myocarditis and Pericarditis after COVID-19 mRNA Vaccines. Queen’s Printer for Ontario. https://www.publichealthontario.ca/-/media/documents/ncov/vaccines/2021/11/myocarditis-pericarditis-mrna-vaccines.pdf?sc_lang=en.

[7] Husby A., Gulseth H.L., Hovi P., Hansen J.V., Pihlström N., Gunnes N., Härkänen T., Dahl J., Karlstad Ø., Heliö T. (2023). Clinical outcomes of myocarditis after SARS-CoV-2 mRNA vaccination in four Nordic countries: Population based cohort study. BMJ Med..

[8] Farrington P., Whitaker H., Weldeselassie Y.G. (2018). Self-Controlled Case Series Studies: A Modelling Guide with R.

[9] Whitaker H.J., Paddy Farrington C., Spiessens B., Musonda P. (2006). Tutorial in biostatistics: The self-controlled case series method. Stat. Med..

[10] Pai M., Chan B., Stall N.M., Grill A., Ivers N., Maltsev A., Miller K.J., Odutayo A., Razak F., Schull M. (2021). Vaccine-Induced Immune Thrombotic Thrombocytopenia (VITT) Following Adenovirus Vector COVID-19 Vaccination. Ontario COVID-19 Science Advisory Table.. Sci. Briefs Ont. COVID-19 Sci. Advis. Table.

[11] Higgins H., Andrews N., Stowe J., Amirthalingam G., Ramsay M., Bahra G., Hackett A., Breen K.A., Desborough M., Khan D. (2022). Risk of thrombosis with thrombocytopenia syndrome after COVID-19 vaccination prior to the recognition of vaccine-induced thrombocytopenia and thrombosis: A self-controlled case series study in England. Res. Pract. Thromb. Haemost..

[12] Weldeselassie Y., Whitaker H., Farrington P. SCCS: The Self-Controlled Case-Series Method. https://CRAN.R-project.org/package=SCCS.

[13] Hawken S. (2023). Time to Detect a Safety Signal in an SCCS Surveillance Analysis. https://stevenhawken.shinyapps.io/sccs_time_to_detect/.

[14] Xavier-Carter B. (2021). Ontario Tops 50,000 Vaccine Doses Administered on One Day for First Time. Toronto Star. https://www.thestar.com/news/gta/ontario-tops-50-000-vaccine-doses-administered-on-one-day-for-first-time/article_ac657ec9-a366-5bc4-b213-4ffe2daa9b48.html.

[15] Pavord S., Scully M., Hunt B.J., Lester W., Bagot C., Craven B., Rampotas A., Ambler G., Makris M. (2021). Clinical Features of Vaccine-Induced Immune Thrombocytopenia and Thrombosis. N. Engl. J. Med..

[16] Fritzell B. (2001). Detection of Adverse Events: What are the Current Sensitivity Limits during Clinical Development?. Vaccine.

[17] Naveed Z., Li J., Spencer M., Wilton J., Naus M., García H.A.V., Otterstatter M., Janjua N.Z. (2022). Observed versus expected rates of myocarditis after SARS-CoV-2 vaccination: A population-based cohort study. CMAJ.

[18] News CBC (2021). “Mild Risk” Prompts Ontario to Recommend Pfizer over Moderna for Those Aged 18–24 | CBC News. CBC. https://www.cbc.ca/news/canada/toronto/covid-19-ontario-september-29-moore-briefing-update-1.6193455.

[19] Barker CIS, Snape MD (2014). Pandemic influenza A H1N1 vaccines and narcolepsy: Vaccine safety surveillance in action. Lancet Infect. Diseases.

[20] Evans D., Cauchemez S., Hayden F.G. (2009). “Prepandemic” Immunization for Novel Influenza Viruses, “Swine Flu” Vaccine, Guillain-Barré Syndrome, and the Detection of Rare Severe Adverse Events. J. Infect. Dis..

[21] Kwong J.C., Vasa P.P., Campitelli M.A., Hawken S., Wilson K., Rosella L.C., Stukel T.A., Crowcroft N.S., McGeer A.J., Zinman L. (2013). Risk of Guillain-Barré syndrome after seasonal influenza vaccination and influenza health-care encounters: A self-controlled study. Lancet Infect. Dis..

[22] CNODES | About CNODES. https://www.cnodes.ca/about/.

[23] Home | Global Vaccine Data Network. https://www.globalvaccinedatanetwork.org/.

